# Sulfur Cycling and Life Strategies in Successional Biocrusts Link to Biomass Carbon in Dryland Ecosystems

**DOI:** 10.3390/microorganisms13112594

**Published:** 2025-11-14

**Authors:** Maocheng Zhou, Qi Li, Yingchun Han, Qiong Wang, Haijian Yang, Hua Li, Chunxiang Hu

**Affiliations:** 1 Key Laboratory of Algal Biology, Institute of Hydrobiology, Chinese Academy of Sciences, Wuhan 430072, China; zhoumaocheng19@mails.ucas.ac.cn (M.Z.); liqi1689@163.com (Q.L.); xjhhyc@163.com (Y.H.); 15927474275@163.com (Q.W.); hjyang@ihb.ac.cn (H.Y.); 2 University of Chinese Academy of Sciences, Beijing 100049, China

**Keywords:** biological soil crusts, carbon-sulfur ratio, cyanobacteria, microbial biomass carbon, microbial network, metagenomics

## Abstract

Examining the changing patterns and underlying mechanisms of soil biomass carbon stocks constitutes a fundamental aspect of soil biology. Despite the potential influence of the sulfur cycle and the life strategies of organisms on community biomass, these factors have rarely been studied in tandem. Biocrusts are model systems for studying soil ecosystems. In this study, metagenomic analysis of biocrusts related to different life strategies from five batches over four consecutive years demonstrated that, in free-living communities, microbial biomass carbon (MBC) synthesis, via assimilatory sulfate reduction (ASR), is primarily coupled with the 3-hydroxypropionate/4-hydroxybutyrate and Calvin–Benson–Bassham cycles. These pathways are affected by the oxidation-reduction potential (Eh), pH, electrical conductivity, and nutrient levels. The decomposition of organic carbon (OC) via dissimilatory sulfate reduction (DSR) was accompanied by the production of dimethyl sulfide (DMS), which was influenced by the C/S ratio and moisture, whereas the synthesis of MBC by symbiotic communities was found to be affected by Eh and pH, and decomposition was affected by the C/S ratio. The MBC stock was influenced by all strategies, with resource strategies having the greatest impacts during the growing season, and the contribution of chemotrophic energy was most significant in free-living communities. In conclusion, the MBC in biocrusts is associated with both ASR and DSR and is facilitated by the A-, S-, and P-strategies under the regulation of the stoichiometric C/S ratio. The exploration of microbial life strategies and sulfur cycling in biocrusts within arid ecosystems in this study offers a new perspective on the patterns of change in soil biomass carbon stocks.

## 1. Introduction

Sulfur is the sixth most abundant element in terms of biomass and serves as the primary energy source for organisms in extreme environments. More importantly, sulfur directly modulates the synthesis and decomposition of organic carbon (OC) in ecosystems because of its high-amplitude oxidation-reduction potential [1,2,3,4], resulting in greenhouse gas emissions and climate change [5]. However, the patterns and mechanisms underlying these effects remain unclear [6,7].

Chemoautotrophic sulfur-oxidizing bacteria are major primary producers that contribute biomass to ecosystems in reduced inorganic sulfur-rich or lightless habitats [8,9,10,11]. In aerobic habitats with high levels of sulfite (SO_3_^2−^) or sulfate (SO_4_^2−^) oxidation, species engage in assimilatory reduction to introduce biomass containing sulfur-containing amino acids into the ecosystem [12,13], while in anaerobic or low-oxygen habitats with high levels of organic matter, microorganisms couple dissimilatory reduction from SO_3_^2−^ or SO_4_^2−^ with OC degradation [14,15], generating H_2_S. The Fe (III) reduction in H_2_S oxidative detoxification further enhances OC decomposition [16,17]. Notably, biomass is mainly influenced by the life strategies of organisms at the community and ecosystem levels. Microbial communities characterized by the K-strategy (in physiological ecology), and Y- and A-strategies (in metagenomics), potentially exhibit high microbial biomass carbon (MBC) contents, while those associated with the r-strategy, and P- and S-strategies, tend to demonstrate low MBC contents [18,19,20,21]. Notably, life strategies have rarely been considered in conjunction with the carbon synthesis and decomposition that are associated with sulfur cycling.

In arid areas, which comprise 41% of the global land area, biological soil crusts (or biocrusts) not only play a role in stabilizing the surface soil and serve as indicators of climate change [22] but also provide a model system for studying soil ecosystems [23]. Although dominant cyanobacteria, lichens, and mosses are the primary producers in these ecosystems, the limited diffusional oxygen transport and local oxygen consumption within biocrusts form a hypoxic microenvironment [24,25]. Additionally, aerobic anoxygenic photosynthetic bacteria that utilize inorganic sulfur are abundant [26,27]. In the context of limited light energy, abundant gaseous energy resources (e.g., H_2_, CO, and CH_4_) [26,28,29,30,31,32], and the microbial community regulating autotrophic and heterotrophic processes via nutrient and carbon availability, we hypothesize that the MBC synthesized by primary producers serves as a foundation for material and energy in the ecosystem and that MBC levels are regulated by the synthesis and decomposition of OC coupled with sulfur cycling. The C/S ratio (the ratio of dissolved organic carbon to reduced inorganic sulfur), which reflects elemental stoichiometry, should also be considered [1,2,3,4,33]. Inorganic sulfur oxidation can supplement light energy in the early stages of oligotrophic succession, with high levels of OC synthesis combined with ASR [34,35]. OC synthesis coupled with organic sulfur oxidation and OC decomposition, combined with DSR or dimethyl sulfoxide (DMSO) reduction, is abundant in the late stage of fertile succession. Most coupled carbon synthesis pathways include the oxygen-resistant Calvin–Benson–Bassham (CBB) cycle and the 3-hydroxypropionate (3HP) cycle [36]. Soil bacteria are generally classified as r-strategists, while soil fungi are K-strategists. It has previously been found that under conditions where the r-strategy predominates early in primary succession (when bacterial communities are dominant) and the K-strategy predominates later (when fungal abundance increases) [37], various communities belonging to the same r- or K-strategy experience changes in resource allocation strategies that may alter the MBC contents on both community and ecosystem scales [19,38] under the joint influence of long-term evolution and short-term domestication [39,40]. For example, the adoption of the stress-tolerant S-strategy might be disadvantageous for MBC accumulation, whereas the A-strategy, which exploits rich inorganic resources, might be beneficial for MBC accumulation. However, when organic carbon becomes the main resource, the A-strategy may instead promote OC decomposition coupled with sulfate reduction, accelerating carbon mineralization and thereby reducing MBC accumulation. Differences in the amount of MBC observed during the growing season are, therefore, probably related to the available resource characteristics [30].

This study is aimed at investigating the associations between microbial life history strategies in biocrusts and MBC synthesis and decomposition while focusing on sulfur cycling. Cyanobacterial and cyano-lichen crusts were selected to signify the early and early-mid stages of ecosystem succession, potentially associated with an r-strategy. Similarly, chloro-lichen crusts and moss crusts were selected to indicate the mid-late and late stages of succession, suggesting an association with a K-strategy. By integrating metagenomic analyses with environmental properties across four consecutive years, this study examines how sulfur cycling and microbial strategies jointly regulate MBC dynamics during biocrust succession. These insights provide a framework for understanding the coupling of biogeochemical processes and microbial strategies in shaping carbon pools in arid ecosystems.

## 2. Materials and Methods

### 2.1. Sample Collection Description

Four biocrust types (cyanobacterial crusts, A; cyano-lichen crusts, C; chloro-lichen crusts, G; and moss crusts, M) were collected annually from the Shapotou ecological restoration region of the Tengger Desert, China (37°32′ N, 105°02′ E) [29,41] from 2015 to 2018. The region is a desert zone that lies between a desert and an oasis and experiences a continental monsoon pattern, with mean annual precipitation of approximately 180 mm, primarily between June and September. Prior observation in the Shapotou region indicated a successional pattern of biocrust communities from A through C and G to M, which is visually distinguishable based on phenotypic traits [42]. Biocrust covers approximately 80% of the soil surface, with 10% A, 50–60% C and G, and 20–30% M [42]. Two batches were collected during June (summer) and September (autumn) 2018 for investigation of the short-term growth effects, and the remaining batches were collected in September of each study year. Each batch comprised all four biocrust types, representing the early (A), middle (C and G), and late (M) successional stages [43]. For each biocrust type, three replicates were collected in 2015, and four replicates were collected in both 2016 and 2017. The 2018 collection covered two distinct growing seasons (June and September), with four replicates collected per season, totaling eight replicates for that year. To ensure temporal continuity across years and balance sequencing depth with representative coverage, one replicate from the 2016 and 2017 collections was selected for metagenomic sequencing. The selected samples represented the most typical phenotypic characteristics of their respective biocrust types (A, C, G, or M). Including all replicates from 2015 and 2018, this targeted selection approach yielded 13 metagenomic samples per biocrust type, ensuring both temporal and successional representativeness.

Sampling was performed when no precipitation had occurred over the previous 72 h. The sampling time was consistently between 10:00 a.m. and 1:00 p.m. To minimize spatial heterogeneity, four plots were selected, each spaced more than 1 km apart. Within each plot, six to eight samples were randomly collected from a 1 m × 1 m area and then mixed to form a single replicate. Biocrust samples (10–20 cm^2^) were carefully removed using a sharp shovel, preserving their natural thickness (4–15 mm) without underlying soil. The samples were immediately placed in sterilized plastic Petri dishes.

### 2.2. Environmental Factor Measurement and Nucleic Acid Extraction

Soil moisture was measured immediately after the samples reached the laboratory. Electrical conductivity and pH were measured in a soil suspension with a soil:water ratio of 1:5 (*w*/*v*), as described previously [41]. Eh was also measured in the same soil suspension using an Eh electrode (mV). Dissolved organic carbon (DOC) was measured using a carbon analyzer (Vario TOC, Elementar, Langenselbold, Germany), MBC was determined using the chloroform fumigation-extraction method [44], and the NO_3_^−^, NO_2_^−^, PO_4_^3−^, and SO_4_^2−^ concentrations were determined using ion chromatography (Thermo Scientific™ Dionex™ ICS-5000, Thermo Fisher Scientific, Waltham, MA, USA). For DNA extraction, obvious bryophytes were removed, and the biocrusts were ground and passed through a 150-µm sieve. Soil microbial DNA was extracted using the PowerSoil DNA Isolation kit (MOBIO Laboratories, Carlsbad, CA, USA) according to the manufacturer’s protocols. DNA purity and concentration were determined using a Nanodrop 2000 (Thermo Fisher Scientific, Waltham, MA, USA), and DNA extraction quality was assessed via 1% agarose gel electrophoresis.

### 2.3. Metagenome Sequencing, Assembly, and Binning

Metagenomic shotgun libraries were prepared according to the manufacturer’s instructions (Illumina Inc., San Diego, CA, USA) and subjected to paired-end sequencing on the Illumina platform [29,41]. Raw reads were trimmed, and quality control was performed using SeqPrep v.1.2 (https://github.com/jstjohn/SeqPrep, accessed on 1 November 2020) and Sickle v.1.33 (https://github.com/najoshi/sickle, accessed on 1 November 2020), using the default parameters. IDBA-UD v.1.1.1 [45] and Newbler (https://github.com/etheleon/newbler, accessed on 1 November 2020) were used for sequence assembly, and contigs (≥300 bp) were predicted using MetaGene v.2.20.0 [46]. A non-redundant gene catalog with 95% identity and 90% coverage was constructed using CD-HIT v.4.6.5 [47], and the gene abundance for each sample was determined by aligning to gene catalogs with 95% identity using SOAP aligner v.2.21 [48] before normalization by gene length.

Biocrusts from the two growing seasons in 2018 were analyzed with high sequencing depth (generating >20 Gb of clean data per sample). Metagenome-assembled genomes (MAGs) were obtained from filtered contigs (>1000 bp) that were processed using the Binning Across a Series of Assemblies Toolkit (BASALT, v1.0.0; parameters: --autopara more-sensitive --refinepara deep) [49]. Genome binning was conducted using MetaBAT2 [50], MaxBin [51], and CONCOCT [52]. The resulting bin sets were dereplicated at 95% average nucleotide identity (ANI) using dRep v2.3.2 [53]. Genome completeness and contamination were evaluated using CheckM v1.0.18 [54] with default settings, and only MAGs with >50% completeness and <10% contamination were retained for downstream analysis.

### 2.4. Functional Annotations and Geochip Analysis

Non-redundant gene catalogs were searched against NR databases (June 2018) using BLASTP (Version 2.3.0; e-value ≤ 1 × 10^−5^). To ensure accurate functional and taxonomic assignments within the microbial community, Bryopsida sequences were excluded at the class level during taxonomic annotation to eliminate plant-derived reads. The remaining sequences were then annotated using the KEGG Orthology (KO) database v59 and BLASTP (e-value ≤ 1 × 10^−5^). The abundance of each KO group was determined by standardizing to the relative abundance of all genes annotated in the KEGG database and multiplying by 1,000,000 [41]; the number of sulfur-cycle-related pathways was determined by calculating the average abundance of the associated KO groups. Relationships observed among the sulfur cycle pathways, marker genes, and KO numbers are listed in Table S1. The taxonomic information for each gene (from the NR database) was integrated with functional data from the KEGG database to ascertain the contribution of each microbial group to the sulfur cycle. Genes predicted from the MAGs were assigned to potential metabolic functions in the main biogeochemical cycles using METABOLIC v4.0 [55]. Geochip 5.0 (180 K) analysis was used to detect the signal strength of marker genes in relation to the sulfur cycle [41].

### 2.5. Classification of Microbial Lifestyle Strategies

To determine the different microbial life strategies, DESeq2 v1.28.1 was used to identify KOs with statistically significant differences (log_2_ fold change > 0, *p*_adj_ < 0.05) for the four types of biocrusts based on negative binomial generalized linear models (Love et al., 2014) [56]. ClusterProfiler v3.16.1 (KEGG database accessed 12 May 2023) was then used to enrich the significant KOs into KEGG metabolic pathways (FDR, *p* < 0.05) [57]. These KOs were subsequently classified into microbial life strategies according to the Y-A-S [19] and Y-A-S-P frameworks [20], based on their functional roles related to yield, acquisition, stress tolerance, and persistence. MBC was used to represent the Y-strategy, in which microbes maximize growth by enhancing their central carbon metabolism and biosynthesis. Genes related to cytochrome oxidase were used to represent aerobic respiration. The life history strategies of the microorganisms in the two growth seasons were then divided using the significant differences in the KOs for June and September. Microorganisms, in which various KEGG pathways were associated with different strategies, were identified by integrating gene classification information (NR database) with functional data from KEGG.

### 2.6. Statistical Analysis

IBM SPSS Statistics 26 (IBM Corp., Armonk, NY, USA) was used to compare the different types of samples (Table S1) using one-way analysis of variance (Tukey’s HSD; *p* < 0.05). Effect sizes (η^2^) were also calculated to indicate the magnitude of between-group differences (Table S1). The psych package in *R* 3.6.2 was used to calculate the Spearman correlation coefficient between genus-level abundances, the main metabolic genes (Table S2), and environmental factors. To explore the positive relationships, the results of positive correlations were retained, and the network was visualized using Gephi 0.9.2 [58] with the Fruchterman–Reingold algorithm layout. Modularity was assessed at a resolution of three. The correlations among MBC, DOC, the MBC/DOC ratio, and life history strategies were calculated using the Hmisc package (version 5.1.0) in *R* 3.6.2. KEGG pathways that exhibited a positive correlation coefficient (r > 0.6) with MBC were selected as representatives for each strategy in the simplified model.

## 3. Results

### 3.1. Sulfur Cycle Patterns

**Figure 1:**
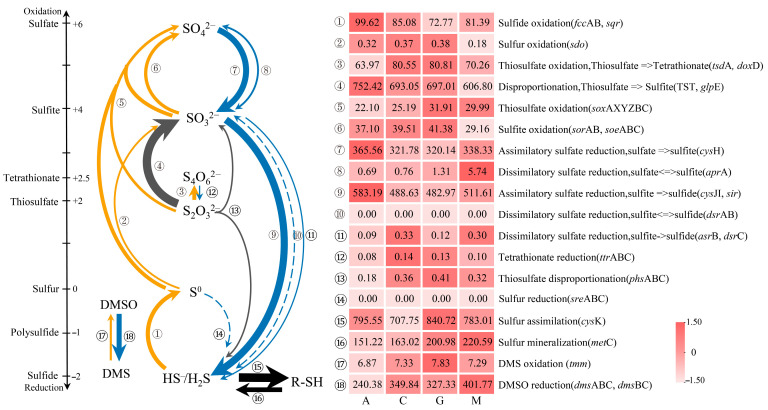
Sulfur cycle in biocrusts across successional stages. Orange, blue, and dark gray arrows indicate oxidation, reduction, and disproportionation reactions, respectively. Dotted lines indicate key genes that were not detected, while the thickness of the solid lines is approximately proportional to the relative abundance of key genes in the pathway, highlighting high-abundance reactions. Values in the heatmap on the right indicate normalized gene abundance after conversion. The successional sequence of biocrust development proceeds from cyanobacterial crusts (A) to cyanolichen crusts (C), chlorolichen crusts (G), and, finally, moss crusts (M).

According to the annotation results (Figure 1), H_2_S oxidation (*sqr*/*fcc*AB) and S_2_O_3_^2−^ oxidation (*dox*D/*tsd*A) were relatively abundant in the oxidation process, with the disproportionation (or oxidation) reaction (TST/*glp*E) being the most abundant in cyanobacterial crusts. In terms of reduction, ASR (*cys*H), assimilatory sulfite reduction (*cys*JI/*sir*), and DMSO reduction (*dms*ABC) were relatively abundant, and DSR (*dsr*ABC/*asr*B/*apr*A) was low. with regard to electron acceptors, quinones (*sqr*/*dox*D/*soe*ABC) were more abundant than cytochromes (*fcc*AB/*tsd*A/*sor*AB) (Table S1).

As succession proceeded, ASR (*cys*H/*cys*JI/*sir*) and high-redox inorganic sulfur oxidation (*fcc*AB/*sqr*/*sdo*) decreased, whereas DSR (*asr*B/*apr*A), DMSO reduction (*dmsABC*), and mineralization (*met*C) increased (Figure 1 and Figure S1).

In terms of the species involved in the sulfur cycle (Figure S2), the disproportionation and sulfur oxidation reactions were mainly associated with actinobacteria and gammaproteobacteria, and the SO_3_^2−^ or SO_4_^2−^ reductions with actinobacteria and alphaproteobacteria. The relative abundance of cyanobacteria decreased significantly with succession, whereas that of planctomycetes increased. Relatively diverse species were found to be involved in DMSO reduction.

### 3.2. Carbon, Nitrogen, and Sulfur Cycling Patterns

**Figure 2:**
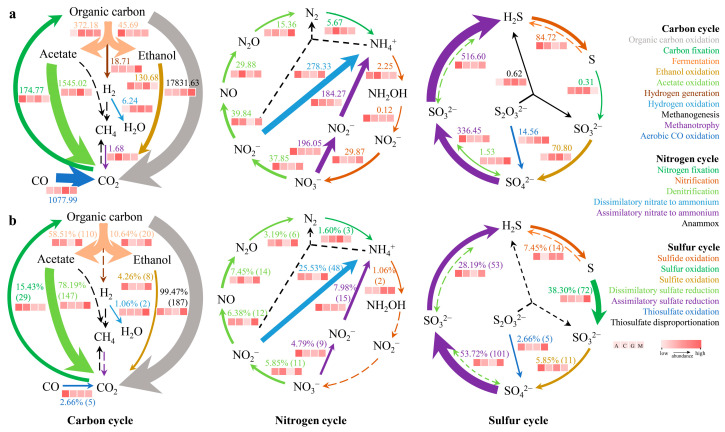
Comparison of carbon, nitrogen, and sulfur cycling in gene catalogs and MAGs obtained for biocrusts. (**a**) Metagenomic non-redundant gene catalogs. Numbers indicate normalized average abundances, calculated as the abundance of each KO group per million total annotated KEGG genes. (**b**) MAGs. Numbers indicate the quantity and percentage of MAGs encoding carbon, nitrogen, or sulfur cycling pathways, with the percentage representing the proportion of total recovered MAGs that contain the respective pathways. Present and absent pathways are indicated by solid and dashed lines, respectively, and the direction of the arrows indicates the direction of the reactions. The thickness of connecting lines reflects the relative abundance across different biogeochemical cycles (C, N, and S), allowing direct comparison among pathways. Heatmaps are individually normalized within each pathway to facilitate comparison of gene abundances across the successional gradient (A, C, G, and M).

A total of 188 medium-quality MAGs (completeness > 50% and contamination < 10%) were recovered by metagenomic binning (Table S3), and the carbon, nitrogen, and sulfur cycling patterns were obtained based on the gene catalog and MAG data (Figure 2). The differences observed were mainly due to issues with the binning technology, which underestimated the CO oxidation in the CO_2_ pathway in the carbon cycles, the oxidation of NO_2_^−^ to NO_3_^−^, the assimilation of nitrate to ammonium (ANRA) in the nitrogen cycle, and the assimilation of sulfite to sulfide in the sulfur cycle, while also overestimating the S^0^ oxidation to the SO_3_^2−^ pathway. Although MAGs provide good coverage of most metabolic pathways in the carbon, nitrogen, and sulfur cycles, the limited sequencing depth and the incomplete recovery of genomes likely led to the under-representation of several key oxidation and reduction reactions. Therefore, subsequent functional and ecological analyses in this study were based primarily on the gene catalog dataset, which provided more comprehensive coverage and higher pathway completeness across biogeochemical cycles. The MAG-based results were retained only for cross-validation to confirm the consistency of overall functional patterns.

### 3.3. Coupling of the Sulfur Cycle with Carbon Metabolism

**Figure 3:**
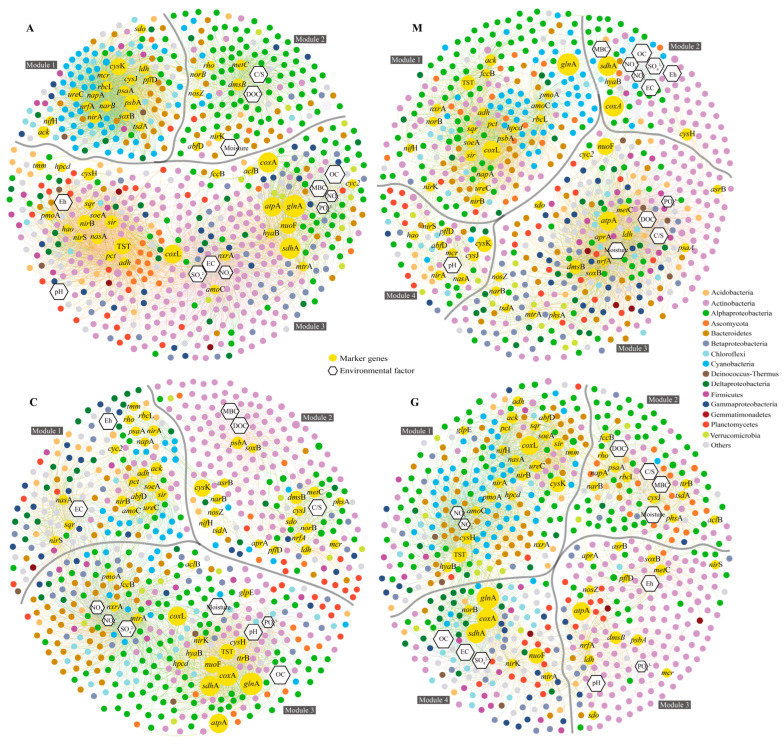
Co-occurrent network depicting highly abundant genera (mean relative abundance >0.01%), major metabolic genes, and environmental factors across each biocrust succession stage. Significance (*p* < 0.01), and positive Spearman correlations are indicated by connecting lines. Nodes are color-coded to represent different phyla or genes, and node size is approximately proportional to relative abundance. Environmental factor nodes are distinguished by a hexagon shape. Modules represent densely connected co-occurrence groups, which were clustered using the Fruchterman–Reingold algorithm in Gephi (modularity assessed at a resolution of 3). Gray lines separate different modules, with each module identified by the “number” in the gray background. A, cyanobacterial crusts; C, cyanolichen crusts; G, chlorolichen crusts; M, moss crusts.

To further understand the relationship between sulfur cycling and carbon metabolism, the main metabolic genes in the carbon–nitrogen–sulfur cycle and environmental factors were correlated with high-abundance genera (average relative abundance > 0.01%) and a co-occurrence network constructed based on the Spearman correlation (*p* < 0.01) (Figure 3). These analyses aimed to identify potential ecological associations. The co-occurrence network analysis revealed a complex but highly modular structure, with the networks for A and C biocrusts organized into three modules each, and those for G and M biocrusts into four modules each. These modules are primarily clustered into two distinct functional categories: synthesis groups and decomposition groups.

Based on the essence of assimilatory and dissimilatory reduction of SO_3_^2−^ or SO_4_^2−^, the synthesis groups of MBC in the four types of biocrusts comprised various inorganic energy sources, electron acceptors, electron donors (fermentation products) with low oxygen reduction potential, and the coupled groups of ASR. CBB cycle (*rbc*L) was the most commonly observed, and the largest groups in all four types of biocrusts showed association with the 3-Hydroxypropionate/4-Hydroxybutyrate carbon fixation cycle (3HP/4HB). Cyanobacteria were abundant and decreased with succession (Figure 3). Decomposition groups comprised dissimilated sulfate-reducing bacteria that were associated with organic sulfur reduction (*dms*B), among which actinobacteria were abundant and increased with succession.

Regarding the environmental factors that were associated with the synthesis and decomposition groups, positive correlations were observed between the early-stage synthesis group [3HP4HB or the reductive tricarboxylic acid cycle (rTCA) (mcr)] and MBC, EC, Eh, pH, and nutrients (NO_3_^−^, NO_2_^−^, SO_4_^2−^), while the decomposition group [Dicarboxylate/4-Hydroxybutyrate Cycle (DC4HB) (*abf*D)] showed a positive correlation with DOC, the C/S ratio, and moisture (A in Figure 3). The late-stage synthesis group displayed positive correlations with MBC, EC, Eh, and nutrients, and the decomposition group exhibited positive correlations with DOC, the C/S ratio, moisture, and PO_4_^3−^ (M in Figure 3). Overall, the synthesis group was primarily involved in processes, namely, assimilatory reduction of SO_3_^2−^ or SO_4_^2−^, inorganic carbon fixation, and fermentation products, exhibiting positive correlations with MBC, EC, Eh, and nutrients. The decomposition group comprised organic sulfur reduction, dissimilatory inorganic sulfur reduction, slight inorganic carbon or heterotrophic carbon fixation, and the decomposition of fermentation products such as lactic acid, with all demonstrating positive correlations with DOC, the C/S ratio, and moisture.

The lichen crusts were comprised not only of a synthesis group that was associated with the assimilatory reduction of SO_3_^2−^ or SO_4_^2−^ pathways and inorganic carbon fixation but also an integral group that was associated with the organic sulfur and inorganic sulfur dissimilatory reduction and 3HP pathways (C and G in Figure 3). The integral group of C was involved in assimilatory SO_3_^2−^ reduction and showed positive correlations with MBC, DOC, and the C/S ratio, whereas the integral group of G did not perform SO_3_^2−^ reduction but was associated with Fe(III) reduction. The synthesis group, composed of CBB or rTCA and associated with S_4_O_6_^2−^ or SO_3_^2−^ reduction, was positively correlated with MBC, DOC, the C/S ratio, and moisture. Therefore, the lichen synthesis group, which also included dimethyl sulfide (DMS) oxidation and S_4_O_6_^2−^ reduction, had no decomposition group but included an integral group that was associated with 3HP. In this context, parallel changes were observed in terms of MBC, DOC, and the C/S ratio.

In addition, the majority of inorganic energy and Fe(III) reduction were observed in the synthesis group during the early stage and the decomposition group in the late stage. Aerobic respiration and H_2_ oxidation, with the largest oxidation-reduction potential difference, were observed in both the early and late synthesis groups, whereas denitrification and the dissimilation of nitrate to ammonium or ANRA were observed in the synthesis and decomposition groups.

### 3.4. Life Strategies

**Figure 4:**
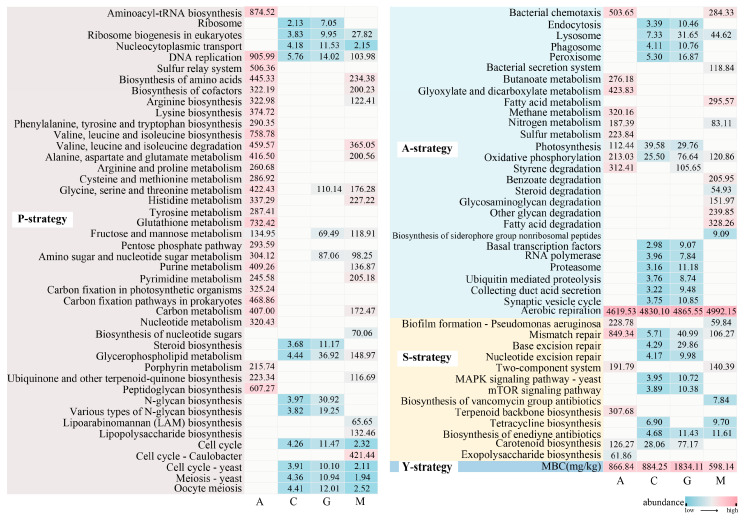
Microbial life strategies at different stages of biocrust succession. P-strategy (cellular and high growth potential maintenance), A-strategy (resource acquisition), S-strategy (stress tolerance), and Y-strategy (high yield) represent different life strategies based on KEGG pathway enrichment analysis. In the heatmaps displaying gene abundance values, the blue/red color intensity represents an abundance gradient, with blue indicating low abundance and red indicating high abundance. A, cyanobacterial crusts; C, cyanolichen crusts; G, chlorolichen crusts; M, moss crusts.

From a succession perspective (Figure 4), early-stage A was characterized by a high level of bacterial chemotaxis and the utilization of inorganic resources; late-stage M was enriched in bacterial extracellular secretion, iron carrier synthesis, aerobic respiration, and OC utilization; middle-stage G showed abundant ribosomes, nucleic acid transport repair, RNA polymerase, polysaccharide synthesis, phagocytic digestion, advanced reproduction, and signal regulation; and C showed similarities to G, but was of relatively low abundance.

**Figure 5:**
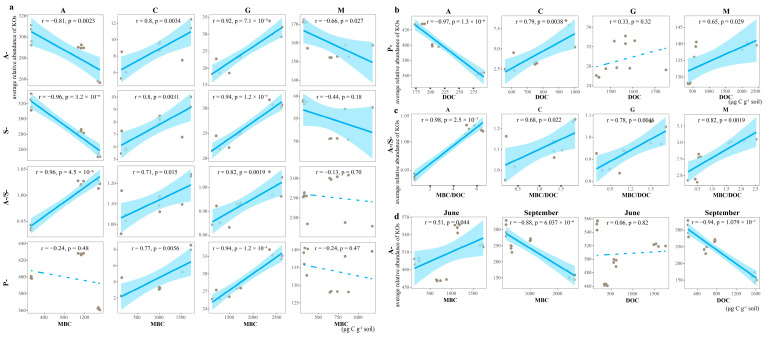
Spearman correlations among different microbial life strategies. Values on the *x*-axis and *y*-axis indicate the mean relative abundance of KOs for each KEGG pathway. Correlation coefficient (r) indicates the strength and direction of the monotonic relationship between variables, and the corresponding *p*-value is used to assess the significance of the correlation. Dotted lines indicate |r| < 0.4. (**a**) Correlation fitting curves between the A-, S-, A-/S-, and P-strategies and MBC. (**b**) Correlation fitting curve between P-strategy and DOC. (**c**) Correlation fitting curve between A-/S-strategy and MBC/DOC ratios. (**d**) Correlation fitting curve for A-strategy during the growth seasons (June and September), MBC, and DOC. A, cyanobacterial crusts; C, cyanolichen crusts; G, chlorolichen crusts; M, moss crusts.

From a correlation perspective (Figure 5), the MBC of the various biocrusts was linked to the A-strategy, and the DOC exhibited a correlation with the P-strategy (except for G). The MBC/DOC ratio was positively correlated with the A-/S-strategy. In summary, the cyanobacterial crusts (A) exhibited A- and S-strategies; the moss crusts (M) exhibited an A-strategy; and the lichen crusts (C and G) exhibited P-, A-, and S-strategies. However, all strategies of C were positively correlated with both MBC and DOC, and all strategies of G were only positively correlated with MBC.

During the growth season (Figure S3), the high MBC contents of A and M were positively correlated with the A-strategy in June; however, the MBC and DOC contents of the lichen crusts (C and G) were negatively correlated with the A-strategy in September (Figure 5d). All biocrusts exhibited an A-strategy during the growing season; however, the MBC of free-living communities was positively correlated with chemical energy acquisition, whereas the MBC of symbiotic communities showed positive promotion with light energy acquisition (Figure S3).

### 3.5. Relationship Between the Sulfur Cycle and Life Strategies

**Figure 6:**
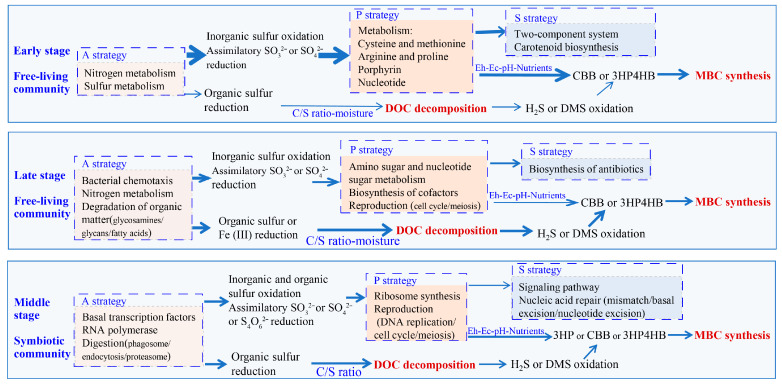
Simplified model showing correlation between key processes in the sulfur cycle, environmental factors, and pathways related to life strategies. Widths of the arrowed lines indicate strength or approximate abundance, while arrows indicate environmental factors influenced by the process. C/S indicates the carbon-to-sulfur ratio, and nutrients include NO_3_^−^, NO_2_^−^, and SO_4_^2−^. Early stage (A); middle stage (C, G); late stage (M). Abbreviations: CBB, Calvin–Benson–Bassham cycle; 3HP, 3-hydroxypropionate cycle; and 3HP/4HB, 3-hydroxypropionate/4-hydroxybutyrate cycle.

Based on the correlation between key processes in the sulfur cycle, the environmental factors, and the pathways related to life strategies (Figure 6 and Figure S4), in the early and late stages of free-living communities, the primary processes were inorganic sulfur oxidation and ASR, driven by light energy and inorganic energy. Moreover, the potential for DSR combined with DOC decomposition was low, and the environmental factors influencing these pathways were similar. However, the early A-strategy was mainly based on the utilization of inorganic energy sources, and the synthesis-related P-strategy was characterized by processes involving sulfur-containing amino acid metabolism, resistance to amino acid metabolism, light energy capture, and individual proliferation. The stress-tolerance of the S-strategy was manifested in its resistance to light radiation and signal response. By contrast, the late A-strategy was primarily based on the utilization of organic resources, and the P-strategy was characterized by efficient and evolved synthetic processes and advanced reproduction. The stress-tolerant S-strategy in the late stage involved antibiotic synthesis, and the proportion of DOC decomposition was higher than it was in the early stage. The potential for sulfur-related synthesis and decomposition was almost equal in the middle-stage symbiotic communities. Additionally, the inorganic and organic energy utilization capabilities of the A-strategy were almost equal. The P-strategy was characterized by efficient evolution and high reproduction, and the expenses of the S-strategy were reflected in various repairs. Additionally, environmental factors such as Eh and pH generally affected synthesis, while decomposition was mainly affected by the C/S ratio.

## 4. Discussion

Early in the ecological evolution of the Earth’s surface, the sulfur cycle was biased toward the oxidation of inorganic sulfur; today, it is biased toward sulfate reduction [59]. In the biocrusts examined in this study, assimilatory reduction of SO_3_^2−^ and SO_4_^2−^ predominated, whereas dissimilatory reduction from SO_3_^2−^ or SO_4_^2−^ was low, which is consistent with the reduction pattern in the current biosphere [59]. The rich metabolic pathways were similar to those commonly observed in microbial mats [60], and the moderate oxidation of SO_3_^2−^ to SO_4_^2−^ was similar to that of groundwater [61,62] and freshwater lakes [63]. The H_2_S oxidation to S^0^ was similar to that observed in ice-capped lakes [64], the deep sea [65], and sediments [66], and the low abundance of DSR was consistent with that of hyper-arid soils [67], contrary to that observed in the deep earth [61]. ASR is an active metabolic pathway in many habitats [68], and the diverse and high potential transformation abilities of S_2_O_3_^2−^ not only reflect its importance in biocrusts [29,69], but also indicate that its chemical form has broad sources and is preferentially transformed by the environment [65,70,71,72], while being economically efficient [13,73,74].

Electron transport proteins with ferricoporphyrin (heme) as the prosthetic group were widely involved in the redox reactions under both aerobic and anaerobic conditions; however, oxidase with quinone was more efficient as an electron medium than cytochrome oxidase with heme iron as the mediator [75]. The biocrust ecosystem favors quinone oxidases over cytochrome oxidases, which suggests higher potential efficiency of sulfur oxidation in the biocrusts and matches the momentarily high production rate associated with light energy utilization [43]. Sulfur metabolism has shaped the composition of the core microorganisms and driven the metabolic interactions within microbial communities [76]. The coexistence of environmental sulfides and oxygen reveals the metabolic diversity of cyanobacteria, which exhibit both oxygenic and anoxygenic photosynthesis [77,78]. The observed organisms and ecosystems offered optimal examples to understand how the environmental and ecological conditions influence primary productivity. Regarding soluble DMSO and volatile DMS, DMSO as a substrate is commonly used in microbial degradation to produce DMS [79]. Both are commonly observed active forms of organic sulfur compounds and free-radical scavengers. The process of DMSO reduction to DMS is an adaptation of the community to high oxidation [80] and potential climate regulation [81,82]. Therefore, the sulfur cycle in biocrusts is characterized by high S_2_O_3_^2−^ oxidation, enriched assimilatory reduction of SO_3_^2−^ or SO_4_^2−^, and low dissimilatory reduction of SO_3_^2−^ or SO_4_^2−^.

Biocrusts are ecosystems with cryptogamic plants as the primary producers, and aerobic anoxygenic photosynthetic bacteria, which utilize light energy, are mainly heterotrophic [83,84]. Because the synthesis group of most OC was associated with CBB and 3HP4HB, and the number of members in the group decreased with succession; thus, it can be inferred that the observed changes in the synthesis group were closely related to changes in the abundances of Cyanobacteria and Archaea [41]. Additionally, fermentation mainly occurred under environmental stress [85], and the OC synthesis that was associated with fermentation end products was mainly composed of CBB and 3HP4HB [4,13], differing from the dark anaerobic deep-earth environment in which the Wood–Ljungdahl pathway predominates [86]. The increasing number of members in the decomposition group, which is characterized by the fixation of a small amount of inorganic carbon and the dissimilatory reduction in DMSO, was consistent with the increasing number of heterotrophic bacteria during succession [29,41,87]. However, the primary decomposition process involved the reduction in DMSO to produce volatile DMS, which differs from the results obtained previously for sediments [88]. Therefore, for the sulfur–carbon relationship within the biocrusts, the decrease in assimilatory reduction of SO_3_^2−^ or SO_4_^2−^ during succession is primarily associated with 3HP4HB and CBB cycles, and the increase in dissimilatory reduction of SO_3_^2−^ or SO_4_^2−^ is accompanied by DMS release.

The sulfur cycle acts as an electron mediator between carbon and nitrate [89]. The synthesis group was positively correlated with MBC, EC, Eh, pH, and NO_3_^−^ and SO_4_^2−^, whereas the decomposition group was positively correlated with DOC, the C/S ratio, and moisture in the early and late stages of biocrust succession (A and M in Figure 3). Therefore, MBC can be considered to represent the result of nitrate and sulfate as electron donors and acceptors for assimilatory reduction within the range of Eh, salinity, and pH, while DOC represents the decomposition of OC under regulation of the C/S ratio and moisture [90,91]. EC and pH, as indicators of the physicochemical properties of the habitats in which electron donors and acceptors reside [92,93,94], significantly influence the distribution and growth of microorganisms. Extensive research has been conducted on the effects that moisture [87], the C/S ratio [33], and Eh [95,96] have on microbial growth. The release of PO_4_^3−^, a crucial nutrient for primary producers [29], from the dissolution of mineral phosphorus [97] due to FeS_2_ formation may affect DOC decomposition [98], leading to the decoupling of the phosphorus, carbon, and nitrogen cycles [99]. Moreover, the oxidation of inorganic sulfur coupled with nitrate reduction is commonly observed in the presence of light, providing electrons for photosynthesis [100,101]. Denitrification produces gaseous nitrogen, and nitrate reduction produces NH_4_^+^. These processes were observed in both the decomposition and the synthesis groups, indicating their primary role as electron donors and acceptors, as opposed to the fixed relationship between MBC synthesis and decomposition [100]. This result supports the regulator view [89] and is similar to that observed in the low-oxygen environments of coastal zones [96]. In summary, the sulfur-associated carbon assimilation is mainly influenced by EC, Eh, pH, nitrate, and sulfate, and carbon decomposition is predominantly affected by the C/S ratio and moisture.

Few studies have investigated the microbial interactions that occur in symbiotic communities [102,103]. However, an integral group is responsible for both synthesis and decomposition in lichen crusts, where heterotrophic and autotrophic microbes have a symbiotic relationship. Moreover, MBC and DOC were found to have positive correlations with the C/S ratio. (C and G in Figure 3). These results indicate that the Ascomycota in cyanolichen crusts, which are predominantly in a state of consumption [39,90,104], are the main contributors to dissimilatory reduction, relying on the autotrophic microorganisms that degrade OC. The Ascomycota in chlorolichen crusts, which are predominantly in an active state [39,90], are the main contributors to ASR and inorganic carbon fixation. Moreover, MBC is positively correlated with Ascomycota, which further demonstrates that the covariance in MBC and DOC is due to the growth of heterotrophic Ascomycota utilizing producer-derived organic substrates, highlighting the trophic coupling between heterotrophs and autotrophs [39]. The nonsignificant impacts of EC, moisture, and nutrients on MBC and DOC in symbiotic communities might be related to their different effects on the biomass of bacteria and fungi [94]. DMS is a critical source of OC, sulfur, and energy [105], and DMS oxidation was observed only in the synthesis group of lichen crusts (C and G), suggesting that it is related to the efficient utilization of DMS as organic energy. The oxidation of S_2_O_3_^2−^ and reduction of S_4_O_6_^2−^ (associated with actinobacteria) occurred only in the lichen crust synthesis group, which may be related to the sulfur supply. In summary, the synchronization of MBC and DOC in the biocrust symbiotic community was associated with 3HP and mixed nutrition and was jointly affected by the C/S ratio. However, cyano-lichen that used the r-strategy tended to decompose, while chloro-lichen crusts using the K-strategy tended to resynthesize the host following the decomposition of the donor product.

Energy drives the transformation of matter in ecosystems. Although light energy drives the formation of biocrusts, this is limited by the environmental conditions, and inorganic energy sources are abundant and readily available, with high energy contents [29,30,31,101,106]. Sources, including various sulfur compounds, ferrous ions (Fe^2+^), H_2_, CH_4_, CO, and NH_3_, were observed in this study (Figure 3). Various inorganic energy sources were associated with 3HP4HB in the early-stage synthesis group, while inorganic energy was observed in the decomposition group during the late stages. This may be because the abundant and persistent inorganic energy [32] is mainly used in the synthesis of OC in the early oligotrophic state [107], while the reduction in organic sulfur and Fe(III) [16] in the late stage is affected by the competition for electron donors, leading to the decomposition of OC and release of DMS. Moreover, the assimilatory reduction of S_2_O_3_^2−^, SO_3_^2−^, or SO_4_^2−^ has been found not only in the synthesis of glutathione pools [108] but also in the synthesis of methionine with high redox potential that involves GlpE [13]. Thus, the inorganic sulfur reduction that changes with succession may have been an adaptive result of organisms synthesizing sulfur-containing amino acids under environmental stress, especially due to the economical nature of S_2_O_3_^2−^ assimilation [13,73]. In addition, algae that produce H_2_ through photosynthesis promote algae-based photolysis [109], and actinobacteria, which may serve as primary producers [26,30], absorb H_2_ from the external environment during periods of starvation to fix CO_2_ [106,110,111]. Thus, the aerobic respiration and hydrogen oxidation that are associated with ASR and MBC may occur simultaneously with MBC synthesis while balancing the oxidation-reduction potential [112,113,114]. These results suggest that both organic and inorganic energy are readily available in biocrusts, with the early stage of succession mainly involving the oxidation of inorganic energy and the coupling of OC synthesis, and the late stage mainly involving the dissimilatory reduction in organic sulfur and iron combined with OC decomposition.

From a life strategy perspective, the r/K framework describes ecological-level trade-offs, where the r-strategy is characterized by rapid growth and reproductive investment, while the K-strategy is characterized by slow growth and stable investment [115,116]. At the genomic and functional levels, these ecological strategies are reflected through the metagenomic Y–A–S–P framework, which captures microbial functional potential rather than population dynamics [20]. However, coexisting communities can overcome resource limitations under oligotrophic conditions [117], and OC levels can alter life strategies [19,38,118]. In this study (Figure 4), two biocrusts (A and C), which were dominated by prokaryotic cyanobacteria, exhibited strong A-strategy characteristics, reflected in the enrichment of amino acid biosynthesis and inorganic energy metabolism pathways that support rapid resource acquisition. Biocrusts G and M were dominated by eukaryotic chloro-lichen and showed enrichment of S- and P-strategy functions, including lysosomal degradation and antibiotic synthesis, indicating enhanced stress adaptation and resource recycling. Therefore, despite being in a late-successional stage, M exhibited the lowest MBC content, suggesting that under relatively nutrient-rich but fluctuating conditions, microbial communities may allocate more energy to maintenance metabolism and turnover rather than net biomass growth. This observation deviates from the classical K-strategy expectation of high MBC but is consistent with our metagenomic evidence that the M is influenced by S- and P-strategy functions (Figure 4) [20]. A, with low MBC content, was characterized by strong stress tolerance and abundant glucose and inorganic energy metabolism [116]—features aligned with S- and A-strategies under low-nutrient conditions [118]. C, which showed higher MBC content than A, was enriched in P-, A-, and S-strategy functions and positively correlated with both MBC and DOC, reflecting a resource-consumptive yet biomass-accumulating stage. G, with the highest MBC content, was enriched in P-, A-, and S-strategy and positively correlated with MBC, which is consistent with the K-accumulation strategy [115,116]. The observation that all types of biocrusts exhibited the A-strategy in both growth seasons (Figure S3) indicates that the growth strategy is determined by the genetic evolution characteristics of the dominant populations [39,119], which is consistent with environmental adaptation [20]. However, the difference in the MBC stock over the various growing seasons (summer and autumn) indicates that MBC accumulation is influenced by the use of growth strategies with different features and branching of the P-strategy [19]. Additionally, the differences in the contributions of chemical and light energy [30] result in high MBC stocks for the free-living communities during seasons with high inorganic energy. In summary, the dominant primary producers in the biocrusts determine the life history strategies of the microbial community. The early stage is rich in terms of inorganic energy utilization and amino acid metabolism while the late stage is rich in regard to organic energy utilization and antibiotic synthesis. From a metagenomic perspective, free-living communities were characterized by the A-strategy, while symbiotic communities exhibited clear A-, S-, and P-strategies. The existing MBC content was affected by all strategies during succession and was only affected by resource utilization during the growing season.

Although the primary producers form the foundation for ecosystem materials and energy, autotrophic synthesis and heterotrophic decomposition are regulated by nutrient availability and accessible carbon sources [120]. Autotrophic cyanobacteria create habitats for heterotrophic microorganisms while adapting to light stress [121]. Moreover, different organisms exhibit different strategies [31,38] depending on the growth conditions [39]. In ecosystem succession, the r- and K-strategies, with different resistance and resilience characteristics, often dominate the early and late stages, respectively [18,122]. In the biocrusts observed in this study, inorganic sulfur oxidation and assimilatory reduction of SO_3_^2−^ or SO_4_^2−^ decreased, and DSR increased as succession progressed. These trends are consistent with reduced carbon fixation and increased nitrate reduction, denitrification, and aerobic respiration [29,41]. Such changes correspond to the variations in the connotations of the characteristic strategies at each stage (Figure 6). Many characteristic metabolic pathways have been discovered [26,123,124], especially in relation to the iron–sulfur cluster cofactors in proteins, such as ferredoxin, and the synthesis of glutathione [125]. The decrease in the MBC/DOC ratio during succession was related to changes in the abundance of cyanobacteria and heterotrophic bacteria [29,41,87] and was positively correlated with the A-/S-strategy ratio (Figure 5). Given that the A-strategy represents resource acquisition and the S-strategy reflects tolerance, this indicates that a higher proportion of carbon is allocated to MBC when the community’s potential for resource acquisition is greater than its investment in stress tolerance. Tolerance consumption was mainly reflected in the DOC and the C/S ratio in the relative abundances of inorganic and organic sulfur resources. Therefore, the characteristic strategies of the dominant population change as the ecosystem evolves, and the existing MBC content in the biocrusts is closely coupled to the assimilation and dissimilation of sulfur, via processes that are realized through the A-, S-, and P-strategies under regulation of the C/S stoichiometry ratio (Figure 6). Nevertheless, several limitations should be acknowledged. This study relied on correlation analyses of metagenomic data, which provide robust evidence of ecological associations and functional potential but do not establish direct causality. In addition, the limited sampling size across five batches over four consecutive years may restrict broader generalization. Future studies integrating metatranscriptomic analyses and larger-scale temporal sampling will be instrumental in validating and refining these findings.

## 5. Conclusions

The conclusions of this study could be summarized as follows: (1) The sulfur cycle in biocrusts is dominated by S_2_O_3_^2−^ oxidation and assimilatory reduction of SO_3_^2−^ or SO_4_^2−^, while dissimilatory reduction of SO_3_^2−^ or SO_4_^2−^ exhibits limited potential. This indicates a primary investment in sulfur for biomass synthesis; (2) biocrusts utilize both organic and inorganic energy, with sulfur and iron oxidation linked to MBC synthesis in the early stage, while DMS production is associated with MBC decomposition in the late stage; (3) in free-living communities, MBC synthesis is linked to sulfur reduction and carbon fixation, and it is influenced by EC, Eh, pH, and nutrients. The decomposition group is driven by dissimilatory sulfur reduction and is influenced by the C/S ratio and moisture. In symbiotic communities, the C/S ratio regulates both MBC and DOC dynamics; (4) free-living communities are characterized by an A-strategy, whereas symbiotic communities exhibit combined A-, S-, and P-strategy traits. These life strategies influence MBC content during long-term succession. Free-living communities correlate with chemical energy acquisition, while symbiotic communities rely on light energy during the short growing season. This study demonstrates that microbial life strategies shaping the sulfur cycle significantly influence the modulation of soil carbon pools within biocrust ecosystems.

## Data Availability

The raw shotgun metagenome sequences in this study are available from the National Center for Bio-technology Information Sequence Read Archive under the accession numbers PRJNA562239, PRJNA548650, PRJNA646196, and PRJNA647185.

## Supplementary Materials

The following supporting information can be downloaded at https://www.mdpi.com/article/10.3390/microorganisms13112594/s1, Figure S1: Normalized sulfur-cycling gene signal intensity in A, C, G, and M; Figure S2: Relative abundance of microorganisms associated with sulfur cycle metabolism genes; Figure S3: Life strategies in biocrusts during the growth seasons (June and September); Figure S4: Microorganisms involved in KEGG pathways of different life strategies in the biocrusts; Table S1: The corresponding relationship of pathways, genes, and KEGG Orthology (KO) number in the sulfur cycle, as well as the variance results across different successional stages; Table S2: The corresponding relationship between the main metabolic genes and pathways in this study; Table S3: The classification and functionality of metagenome-assembled genomes (MAGs).
