# Supplementary material for: Sulfur Cycling and Life Strategies in Successional Biocrusts Link to Biomass Carbon in Dryland Ecosystems
Source: Microorganisms. 2025 Nov 14;13(11):2594. doi: 10.3390/microorganisms13112594 (PMC12654827; doi:10.3390/microorganisms13112594)
Supplement: Supplementary file 1 [file microorganisms-13-02594-s001.zip › microorganisms-3899592-supplementary/Supplementary Figure.pdf]

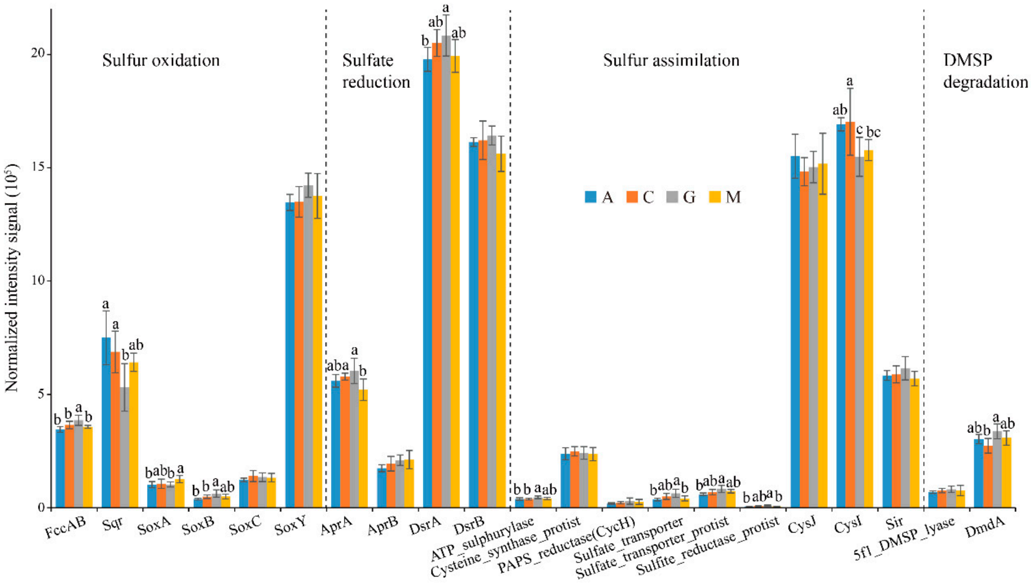

Figure S1. Normalized sulfur-cycling gene signal intensity in A, C, G, and M. Genes are grouped into four categories: sulfur oxidation, sulfate reduction, sulfur assimilation, and DMSP degradation. Significant differences among sample types, determined using the Wilcoxon rank-sum test, are indicated by the letters a, b, and c. Data represent the mean values and standard deviations. Different letters indicate P < 0.05. A, cyanobacterial crusts; C, cyanolichen crusts; G, chlorolichen crusts; M, moss crusts.

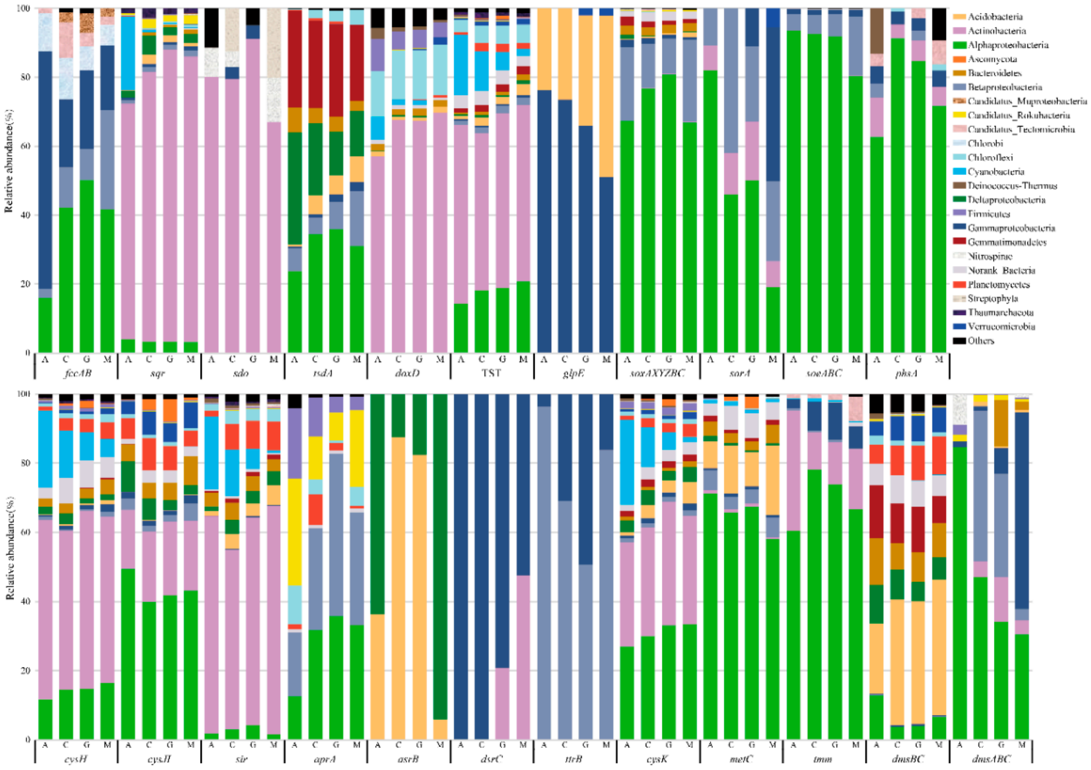

Figure S2. Relative abundance of microorganisms associated with sulfur cycle metabolism genes. A, cyanobacterial crusts; C, cyanolichen crusts; G, chlorolichen crusts; M, moss crusts.

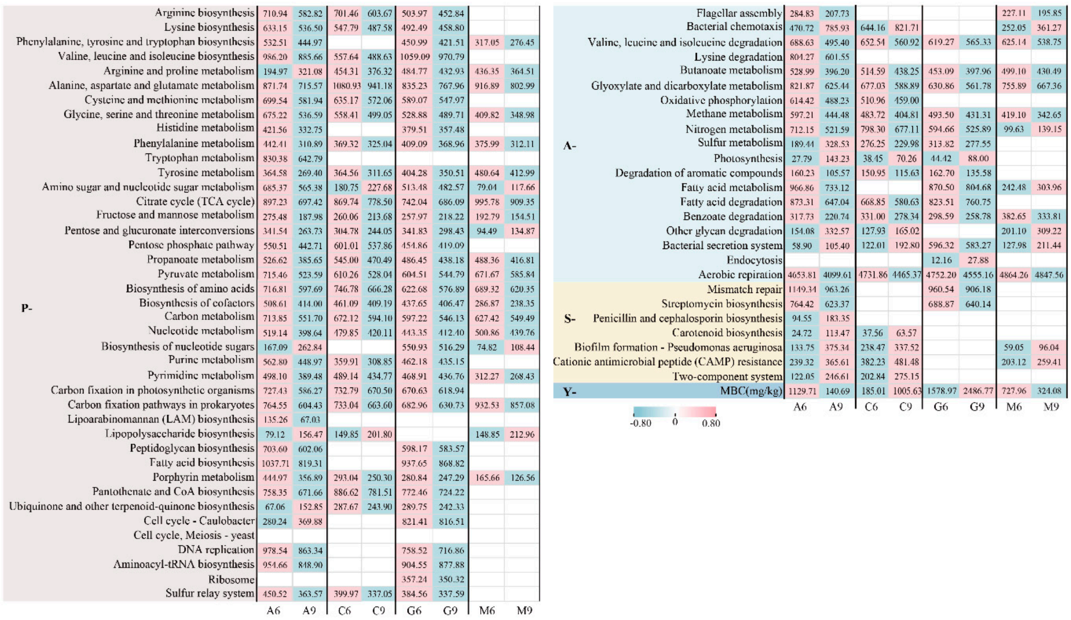

Figure S3. Life strategies in biocrusts during the growth seasons (June and September). P-, cellular and high growth potential maintenance strategy; A-, resource acquisition strategy; S-, stress tolerance strategy; Y-, high yield strategy. A6/9, cyanobacterial crusts collected in June/September; C6/9, cyanolichen crusts collected in June/September; G6/9, chlorolichen crusts collected in June/September; M6/9, moss crusts collected in June/September.

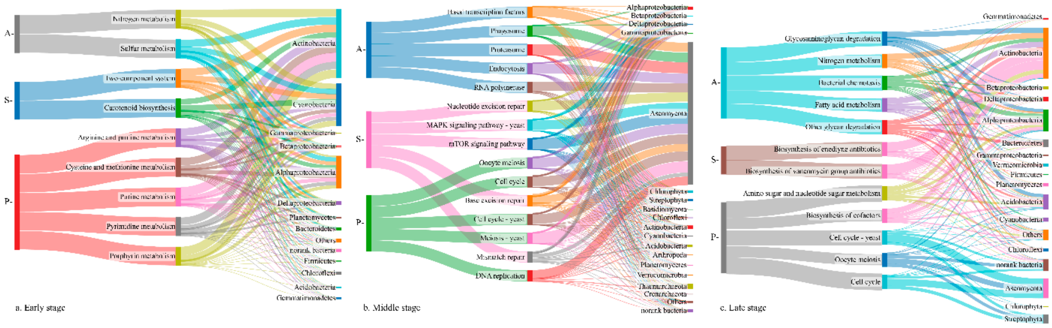

Figure S4. Microorganisms involved in KEGG pathways of different life strategies in the biocrusts. early stage, cyanobacterial crusts; middle stage, cyanolichen crusts and chlorolichen crusts; late stage, moss crusts. P-, cellular and high growth potential maintenance strategy; A-, resource acquisition strategy; S-, stress tolerance strategy.
